# Implementation of medication reviews to optimize the use of medications in Swiss nursing homes: a mixed-methods study

**DOI:** 10.1186/s12913-025-13042-8

**Published:** 2025-07-08

**Authors:** Stephanie Mena, Julie Dubois, Marie Schneider, Anne Niquille

**Affiliations:** 1 Department of Policlinics, Community Pharmacy, Unisanté, Center for Primary Care and Public Health, University of Lausanne, Lausanne, Switzerland; 2 School of Pharmaceutical Sciences, University of Geneva, Geneva, Switzerland; 3 Institute of Pharmaceutical Sciences of Western Switzerland, University of Geneva, University of Lausanne, Geneva, Switzerland; 4 Faculty of Science and Medicine, Institute of Family Medicine, University of Fribourg, Fribourg, Switzerland; 5 Department of Epidemiology and Health Systems, Center for Primary Care and Public Health (Unisanté), University of Lausanne, Lausanne, Switzerland

**Keywords:** Medication reviews, Deprescribing, Implementation Science, Nursing homes

## Abstract

**Background:**

Polypharmacy, can cause drug related problems (DRPs), including the use of potentially inappropriate medications (PIMs). Services such as medication reviews (MRs) have been initiated to address DRPs and PIMs, but their implementation remains underreported. In 2021 and 2022, a pilot project was developed with the goal of reorganizing the care of a pharmacy service to introduce a patient-centered, interprofessional MR service. The project, Medication Reviews in Nursing Homes (MRNH), took place in 10 Swiss nursing homes (NHs). The aim of this study was to evaluate the implementation and impact of the MRNH project in order to gain a better understanding of the processes involved in implementing MR in nursing homes.

**Methods:**

This observational study followed a Type 3 hybrid implementation-effectiveness design using quantitative and qualitative analyses. Relevant implementation outcomes were defined through the Framework for the Implementation of Services in Pharmacy (FISpH) and the Reach, Effectiveness, Adoption, Implementation, Maintenance RE-AIM framework. Data were collected via questionnaires, focus groups and administrative records. The study evaluates factors and strategies related to the implementation of MRs and assesses the impact of MRs based on the proportion of resolved DRPs at a four-month follow-up.

**Results:**

The target was for each NH to perform MRs for 10% of the NHs population. Seven of the NHs achieved this goal, which results in 55 MRs presented of a theoretical total of 75. Following interprofessional team discussions, treatment plans including 145 modifications were created, of which 128 were effectively implemented. As 120 of them were maintained at follow-up, MRs performed lead to a 83% of a partial or complete resolution of the DRPs detected (CI: 74.5–90.7%; 43 MRs). Implementation strategies were considered as useful by HCPs, including pharmacist training, clinical support and audit & feedback and defining their own sub-process. Seven of 10 participating NHs continued MRs after MRNH.

**Conclusions:**

The implementation of MRs in NHs was successful in seven of the 10 participating NHs. The service was considered feasible and accepted, and its dissemination was recommended by the participating healthcare professionals. The results of the study support the decision of the regional health department to extend the service to more NHs and may help identify strategies to further sustain its implementation.

**Supplementary Information:**

The online version contains supplementary material available at 10.1186/s12913-025-13042-8.

## Background

The ageing population represents significant challenges to healthcare systems due to the increased prevalence of frailty, multimorbidity, and polypharmacy [1]. The overall prevalence of polypharmacy, defined as the concomitant use of five or more medications [2], among older adults (aged 65 years or more) has been estimated to be 45% [3], a proportion similar to the 46% found among the Swiss nursing home (NH) residents [4]. However, polypharmacy has been associated with negative outcomes, including falls, hospitalizations, and mortality, especially among older adults [2], and can increase the risk of drug related problems (DRPs) [5, 6], i.e. “events involving drug therapy that actually or potentially interfere with desired health outcomes” [7]. The use of potentially inappropriate medications (PIMs) is a particular cause of DRP related to the use of medications with risks that outweigh their benefits [8]. PIMs have been shown to affect the health and quality of life of older people and to increase the costs of the healthcare systems [9, 10].

Medication Review (MR), which refers to a “structured evaluation of a patient’s medicines to optimize medicines use and to improve health outcomes” [11], has often been proposed to address polypharmacy and PIMs [12]. However, the use of MRs has had mixed results [13–15]. A recent Cochrane systematic review concluded that such interventions may have little or no effect on improving clinical outcomes, such as hospital admissions or quality of life, but may lead to some improvements in intermediary outcomes, such as medication appropriateness and number of PIMs [12]. These findings indicate the need for further investigation into whether such mixed results are due to ineffective interventions or poor implementation. In addition, evaluating implementation provides an opportunity to better understand the factors that lead to successful or unsuccessful implementation [12, 14, 16–19]. The implementation of MR services in NHs has not yet been thoroughly evaluated; thus, there is a need to further describe and test the implementation of interventions and strategies to optimize pharmacotherapy for older adults in NHs [20].

Implementation science provides frameworks and methodology to implement an intervention in a structured way by helping researchers define and design implementation projects [21, 22]. It is fundamental to develop and evaluate implementation strategies, which can be defined as “methods or techniques used to enhance the adoption, implementation, and sustainability of a clinical program or practice” [23]. Training, audit, and feedback, and incentives are well-documented implementation strategies [20].

This present study aims to test implementation as well as assess the impact of MRs on DRP resolution conducted within a pilot project, called Medication Review in Nursing Homes (MRNH), developed to implement advanced MRs’ service in 10 NHs of the canton of Vaud in 2021 and 2022.

## Methods

### Design

This implementation and impact study used a type 3 hybrid implementation design [23] comprising both quantitative and qualitative methods.

### Study context

In 2009, the Swiss canton of Vaud implemented an integrative pharmacy service to optimize medication use in NHs. The service uses the quality circle methodology [24], and involves nurses, pharmacists, and physicians. Regular meetings are held between representatives from each of these healthcare professions, during which local drug prescribing recommendations are developed based on the latest scientific evidence and NH consumption data. These recommendations are reevaluated annually.

As the integrated pharmacy service in place did not specifically or efficiently address the prevalence of PIMs at the NH level [25], the regional health authorities decided in 2021 to fund a pilot project intitled Medication Review in Nursing Homes (MRNH). The aim of MRNH was to develop and implement an advanced medication review (MR) within the existing service.

MRNH was a follow-up to a study [25], where quality circle meetings on deprescribing and MRs were consecutively trialed in voluntary NHs to test their effectiveness on deprescribing PIMs and improving patient safety [26, 27]. Based on this previous experience as well as implementation observations, the research team defined six implementation strategies to support MR services in 10 NHs. An evaluation of the implementation and impact of MRs was conducted in parallel to gain a better understanding of the process in the context of the pharmacy service in place and to facilitate the sustainable integration of MRs into the care routines of a larger number of NHs, if effective. Thus, the main objectives of this impact and implementation study were 1) to evaluate the implementation, especially the implementation strategies, of MRs and identify determinants, and 2) to assess the impact of MRs on the proportion of resolved DRPs at four-month follow-ups.

### Study population

- Nursing homes

The MRNH pilot study was designed to include 10 voluntary NHs with a geriatric and/or psychogeriatric mission that had been active in the integrated pharmacy service for at least three years at the time of the recruitment. Each NH team consisted of physicians, NH-referring community pharmacists, and nurses. The NHs were recruited via an e-mail sent to members of the local professional associations of pharmacists, physicians, and NHs in October 2020. Regular reminders to participate were sent via email during the following four weeks. Before the project’s start, the director, head nurse, referring physician, and pharmacist of each NH had to sign an agreement form, committing to participate in the pilot project.

- Residents

Each NH team selected 10% of their residents based on minimal inclusion criteria in addition to their own criteria, chosen to provide MRs to the residents who will benefit the most. Minimal criteria were the following: 75 years or older NH resident admitted for long stay, with a five or more regular medications intake and an estimated life expectancy of more than six months. The nurses informed those residents or their legal representative (in case of cognitive impairment) about the pilot project and asked them to sign a consent form allowing the use of their personal medical data for the observational study.

### MR process

**Fig. 1:**
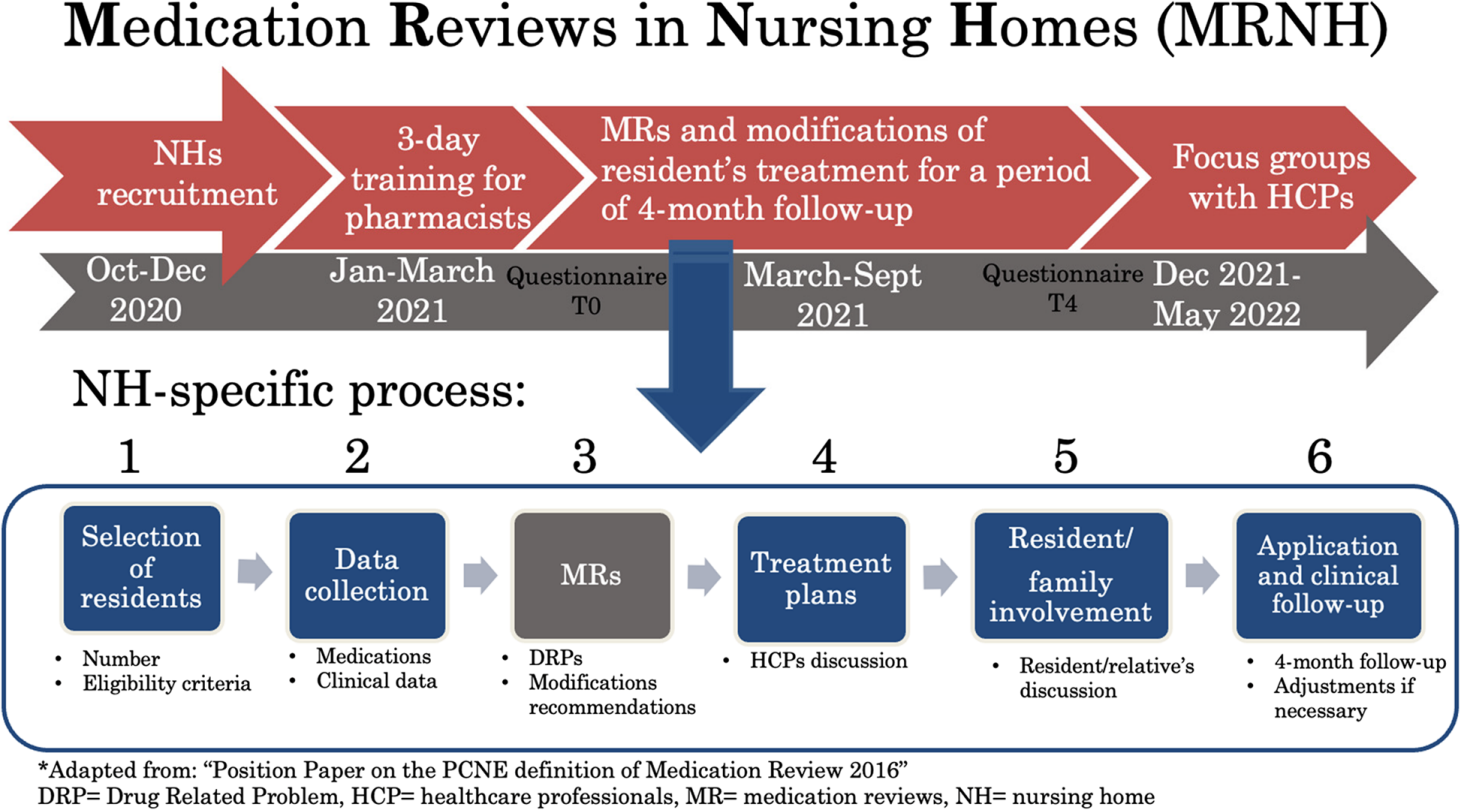
MRNH process and timeline. I Adapted from “Position Paper on the PCNE definition of Medication Review 2016”. DRP = drug related problem, HCP = healthcare professionals, MR = medication reviews, NH = nursing home

Referring nurses were responsible for providing clinical data about the selected residents, including their diagnosis, laboratory values, living preferences and treatment goals. The community pharmacists referring for the NHs led an advanced MR [11] to identify DRPs and propose treatment modifications, such as formulation, frequency, dosage, duration, therapeutic switch, or deprescribing. These recommendations were then discussed with the referring nurses and physicians to establish treatment plans, using a standardized template provided by the research team. Those plans were communicated to the residents or their representatives. Follow-up occurred four months after the first treatment modification, with individual monitoring specified in the treatment plans. Safety of the service was monitored by tracking hospitalizations, falls, use of physical restraints, and deaths. Figure 1 details the MRNH pilot study process and timeline.

### Description of implementation strategies

The six pre-identified implementation strategies, categorized according to Powell et al.’s [28] and Perry et al.’s taxonomies [29], were as follows: 1) pharmacist training, 2) financial incentives for nurses and pharmacists, 3) clinical support from a local pharmaceutical assistance unit (i.e., pharmaceutical support provided by the university center of primary care and public health (Unisanté) where the research study was carried out via telephone or e-mail to all pharmacists who requested it), 4) audit and feedback through monitoring, 5) adaptation of the specific steps of the MRNH process by each NH team, and 6) an online platform to enhance exchanges between participating pharmacists. These strategies are detailed below.

1. The first implementation strategy was a pharmacist training workshop that was designed specifically for the project and corresponded to the preparation phase. The duration was three days that were spread over three months (one day of training per month). The workshop covered the following topics: 1) clinical reasoning and how to conduct and perform MRs with existing tools, 2) interprofessional communication, and 3) how to conduct the project with the materials provided. The latter consisted of a checklist to assist the pharmacists in the implementation of the project and a template for structuring MRs and establishing treatment plans, including causes of DRP coding, as well as documenting residents’ clinical follow-ups.
2. The second implementation strategy involved providing financial incentives to nurses and pharmacists; these were granted by the regional health authorities. The incentives amounted to one hour of work for nurses and two hours of work for pharmacists per MR. In addition, pharmacists were granted an extra three hours per active NH to implement the MR process and collect and transmit data to the research team. Physicians were remunerated through the healthcare insurance system based on a tariff for medical services, defined by the Swiss Federal Office of Public Health [30]. This system allows physicians to bill health insurers directly for services provided to residents.
3. The third implementation strategy involved providing clinical support through the pharmaceutical assistance unit of Unisanté. If needed, pharmacists could ask this unit to perform one or several MRs in exchange for one hour of remuneration per MR, but they had to provide all the data and lead the discussion to reach the treatment plan.
4. The fourth implementation strategy was audit and feedback, which were provided through monitoring. A PhD student (SM) followed up regularly with the referring pharmacists via email or phone call to monitor the NH teams’ progress regarding compliance with deadlines, number of MRs performed, and need for extra clinical support.
5. The fifth implementation strategy was to actively involve the NH teams by asking them to define and document their processes at each step (see Fig. 1). This strategy included defining the role and task by profession at each stage.
6. The sixth implementation strategy involved the use of an online chat platform to facilitate experience-sharing between pharmacists. The chat was established on Slack™ by the research team, who also used it to suggest specific tools to perform MRs and share other literature and clinical references.

### Data collection and analysis

For the evaluation of the implementation outcomes and strategies, data were collected via questionnaires, focus groups, and MRNH and IPS monitoring (detailed in Table 1). These involved documenting the progress and implementation of MRNH.

**Table 1:** Implementation strategies, implementation outcomes, data source, and associated measures

**Implementation strategy**	**Category **	**Measure**	**Data source**
Three-day training workshop for pharmacists	Conduct educational meetings for pharmacists: training for pharmacists	Training evaluation: satisfaction (scale 0 = minimum–5 = maximum), adequacy content (5-point Likert scale, from very useful to not useful at all*), barriers and facilitators (free text)	Questionnaire T0, focus groups
Financial incentives for pharmacists and nurses	Finance strategies	Financial adequacy of service perceived by the three HCPs (yes/no for questionnaires)	Questionnaire T4, focus groups
Clinical support for pharmacists	Ongoing consultation	• Utility perceived of the support provided to pharmacists (5-point Likert scale, from very useful to not useful at all*)• Number of participating pharmacists who asked for clinical support	Questionnaire T4, MRNH monitoring
Monitoring NH team’s progress	Audit and feedback	Utility perceived of the monitoring by HCPs (5-point Likert scale, from very useful to not useful at all*)	Questionnaire T4
Definition of MR specific implementation processes for each NH	Prepare HCPs to be active participants of the change	• Utility of defining NH specific process before the implementation of the service by HCPs (5-point Likert scale, from very useful to not useful at all*)• Degree of involvement of HCPs at each stage (5-point Likert scale, 1 = minimum–5 = maximum),	Questionnaire T4
Online platform for pharmacists	Create online learning communities	Utility perceived by pharmacists (5-point Likert scale, from very useful to not useful at all* for questionnaires)	Questionnaire T4, focus groups

**Stages of implementation**	**Implementation outcome**	**Measures**	**Data source**
*Preparation*	Adoption	• Number and representativeness of participating NHs• Number of NHs teams who signed the agreement form to participate before the beginning of the project	MRNH monitoring
*Operation*	Reach	• Proportion of residents having received a MR• Reasons for not reaching the expected number of MRs performed (free text)	MRNH monitoring
	Acceptability	HCPs satisfaction of the new service on a 5-point Likert Scale, ranging from very satisfied to very dissatisfied	Questionnaire T4
	Feasibility	Availability of resources (time, finances, staff, skills) for pharmacists on a 5-point Likert scale, from strongly disagree to strongly agree; validated questions from [31]	Questionnaire T0 for skills and T4 for other resources
	Implementation	• Adaptation of each NH processes (free text): o NH residents’ selection o Data collection to perform MR o Conducting MR o Treatment plans implementation o Involvement of NH resident/family in the MR process o Follow-up of treatment changes• Barriers and facilitators to implement the new service and specific ones at each step of the MR process (pharmacist’s perspectives; free text)	MRNH monitoring, processes documented with the template provided + HCPs focus groups + Questionnaire T4 (time required for HCPs to perform MR by each step) Free text from questionnaire T4 + HCPs focus groups
	Fidelity	• Average time required to complete MRs for pharmacists• Number of NHs with defined specific process• Number of NHs that reached the number of MR defined at baseline (10% of the NH residents)• Proportion of NHs in the schedule planned and reasons of delay if applicable• Number of NHs who filled the treatment plan’s template provided	Questionnaire T4 MRNH monitoring Questionnaire T4 Questionnaire T4 + MRNH monitoring MRNH monitoring
*Sustainability*	Perspectives on maintenance	• Proportion of HCPs who would recommend the service to other NHs• Number of HCPs who intend and proportion of HCPs who would find useful to reconduct MRs• Proportion of NHs that renewed the new service after the project	Questionnaire T4 and HCPs focus groups Questionnaire T4 Pharmacy service monitoring

*HCP* Health care professional, *MR* Medication review, *MRNH* Medication review in nursing home, *T0* Baseline, *T4* Four-month follow-up, *NH* Nursing home

*A combination of very useful and useful has been applied to show the results

#### Quantitative data

Questionnaires were administered at two points: one to pharmacists only after the training (i.e., at baseline [T0], see Appendix 1), and the second to all healthcare professionals (HCPs) after the four-month follow-ups (T4). The T4 questionnaire, which is presented in Appendix 2–4, comprised a specific version for each profession and was administered during September and November 2021. The T0 questionnaire was based on three domains of the Framework for the Implementation of Services in Pharmacy (FISpH): 1) skills, 2) training, and 3) process of the service. The T4 questionnaire also included three main elements: 1) evaluation of the implementation strategies, 2) evaluation of the implementation outcomes, and 3) facilitators and barriers to the implementation of the service. In the T4 questionnaire, the pharmacists were asked to provide a detailed assessment of the determining factors at each stage of the MR process. In addition, all HCPs were asked to document the time required for the MR process. These were expressed as minimum and maximum times (in minutes). Pharmacists were also asked to report the duration of interprofessional discussions to validate the treatment plans resulting from the MRs. The questionnaires were reviewed by a researcher from Unisanté who was not directly involved in the project to ensure their comprehensiveness. Table 1 shows the quantitative data collected. Other relevant data came from the routine monitoring of the integrated pharmacy service and included characteristics of the NHs (e.g., number of beds, mission, location, year of pharmacy service entry). Proportion, means and standard deviation were used to analyze the data.

#### Qualitative data

After the T4 questionnaires were collected, three focus groups (one per profession), whose interview guides are presented in Appendix 5–7, were organized between December 2021 and May 2022. An invitation was sent via e-mail to all participating HCPs, whose contact information had been collected through the MRNH monitoring at the beginning of the project. For HCPs who were not able to join the focus groups, semi-structured individual online or face-to-face interviews were offered. The topic guide used to lead both the interviews and the focus groups was based on a preliminary analysis of the barriers and facilitators identified with the T4 questionnaires. Another element of the guide concerned maintenance (i.e., the intention to continue the service). Oral consent was obtained before the interviews began. Each focus group was led by a qualitative researcher (JD) and a PhD student (SM), who took turns leading the groups and taking notes. Face-to-face focus groups and interviews were audio recorded. Online focus groups and video interviews were recorded using the Zoom online platform. All audio files were transcribed using Trint™ software. To ensure that the transcriptions were correct, a research assistant proofread the transcripts while listening to the corresponding audio recordings.

Interview transcripts were coded with the assistance of MAXQDA AnalyticsPro software (2022) using thematic analysis [23] and a primarily deductive approach. As a first step, one transcript was coded separately by two researchers (SM and JD), based on the interview guide. The results were compared and discussed to reach agreement over which codes to use and their definitions. The resulting codebook was then applied by SM to all remaining focus groups and interviews. When subjects not covered by the interview guide were identified, they were coded using an inductive approach. The codebook was adapted iteratively as new codes emerged from the data. New codes and changes in the definitions of codes were discussed between JD and SM for agreement. Regular cross-checks of the coding were performed by JD to ensure the validity and rigor of the process. After both authors agreed on the coding, all codes were compared, and similar codes were merged and combined into sub-themes and themes. Transcripts from nurses, pharmacists, and physicians were first coded separately. Themes were grouped according to their similarities where relevant.

### Implementation evaluation

#### Strategies

The actual use of each strategy is described quantitatively. The HPCs’ satisfaction with the strategies was assessed both quantitatively (5-point Likert scales; questionnaire T4) and qualitatively (focus groups).

#### Outcomes

Implementation outcomes [22] were defined using the FISpH [32] and the Reach, Effectiveness, Adoption, Implementation, Maintenance (RE-AIM) frameworks [33]. Proportions were calculated for adoption, reach, acceptability, feasibility, fidelity, and maintenance.

#### Impact evaluation

To evaluate the impact of MRNH, the following measures were used: 1) the percentage of DRPs considered to be resolved at the four-month follow-up, and 2) the number and mean of DRPs identified by pharmacists per resident. DRPs were coded by the pharmacists according to the PCNE classification for DRPs V9 [7] for problems, causes, intervention acceptance, and maintenance at follow-up. Safety indicators (hospitalization, falls, deaths, and use of physical restraints) were recorded through electronic case report forms using the REDCap database. In case of the death of a resident, NH teams were asked to complete a serious adverse event (SAE) report to determine the causal link between the death and the deprescribing (1 = unrelated–5 = definitely related). The proportion of resolved DRPs and its 95% confidence interval were estimated via a three-level mixed-effect logistic regression, with DRP nested within resident and resident nested within NH. This model considered the lack of independence due to multiple DRPs assessed per resident and to multiple residents within each NH. Data were analyzed using the statistical package Stata (Stata Corp, 4905 Lakeway Dr., College Station, TX 77845, USA) software. The impact of each MR was also assessed through the T4 questionnaire, which gathered nurses’ and physicians’ perceptions of residents’ condition at follow-up based on four elements: 1) physical well-being (pain, functional autonomy, ability to move, etc.), 2) psychological well-being (morale, mood, etc.), 3) state of awareness during the day (energy, dynamism, etc.), and 4) quality of exchanges (interaction, trust, etc.). For each of these elements, the nurses and physicians were asked whether the element had been impacted positively, negatively, not at all, or in some other way.

### Reporting

Standards for Reporting Implementation Studies (STARI) [34] guidelines were used to structure this article.

## Results

### Evaluation of implementation strategies

#### Pharmacist training

The pharmacists were satisfied with the training provided; they rated its quality as mean ± SD of 3.7 ± 0.7 out of 5 (*n* = 9). The content of the course generally met the expectations of the pharmacists (*n* = 7/10). Regarding the adequacy of the training, five pharmacists found it sufficient to perform MRs.

In the T0 questionnaire, the pharmacists mentioned the following strengths of the training: 1) interprofessional workshops, 2) discussion and exchange between participants, and 3) a detailed explanation of the stages of the project, including a timetable. They also identified several weaknesses: 1) virtual training due to the Covid pandemic, 2) the lack of an intervention from an MR specialist, and 3) the lack of specific clinical guidelines. Some pharmacists appreciated the clinical case-solving session as it was presented, while others wanted additional cases. During the focus groups, several pharmacists reiterated the desire for an intervention from an MR specialist during training and the provision of clinical guidelines, and they underlined the necessity of interpreting laboratory values, a dictionary of common medical abbreviations, and a list of the most common pathologies in psychogeriatrics.

#### Financial incentives

Most of the pharmacists (*n* = 7/10), two nurses (*n* = 2/7), and three physicians (*n* = 3/8) thought they were insufficiently remunerated for the service delivered, while one nurse and one physician considered the remuneration to be sufficient. The others did not express an opinion on this matter. In the interviews, the physicians stated that the billing position related to consultations in the absence of a resident, limited to one hour per trimester, did not provide adequate compensation.

#### Clinical support for pharmacists

Three pharmacists requested clinical support to help them perform their MRs (*n* = 3/10), while three other pharmacists asked for feedback on their MRs. At follow-up, three pharmacists mentioned that the clinical support was useful or very useful, three found it not useful, and one did not express an opinion.

#### Monitoring NH teams’ progress

Among the nine responding pharmacists, seven found the monitoring through audit and feedback useful. Two pharmacists did not express an opinion, and one did not find it useful.

#### Definition of specific MR implementation processes for each NH

**Fig. 2:**
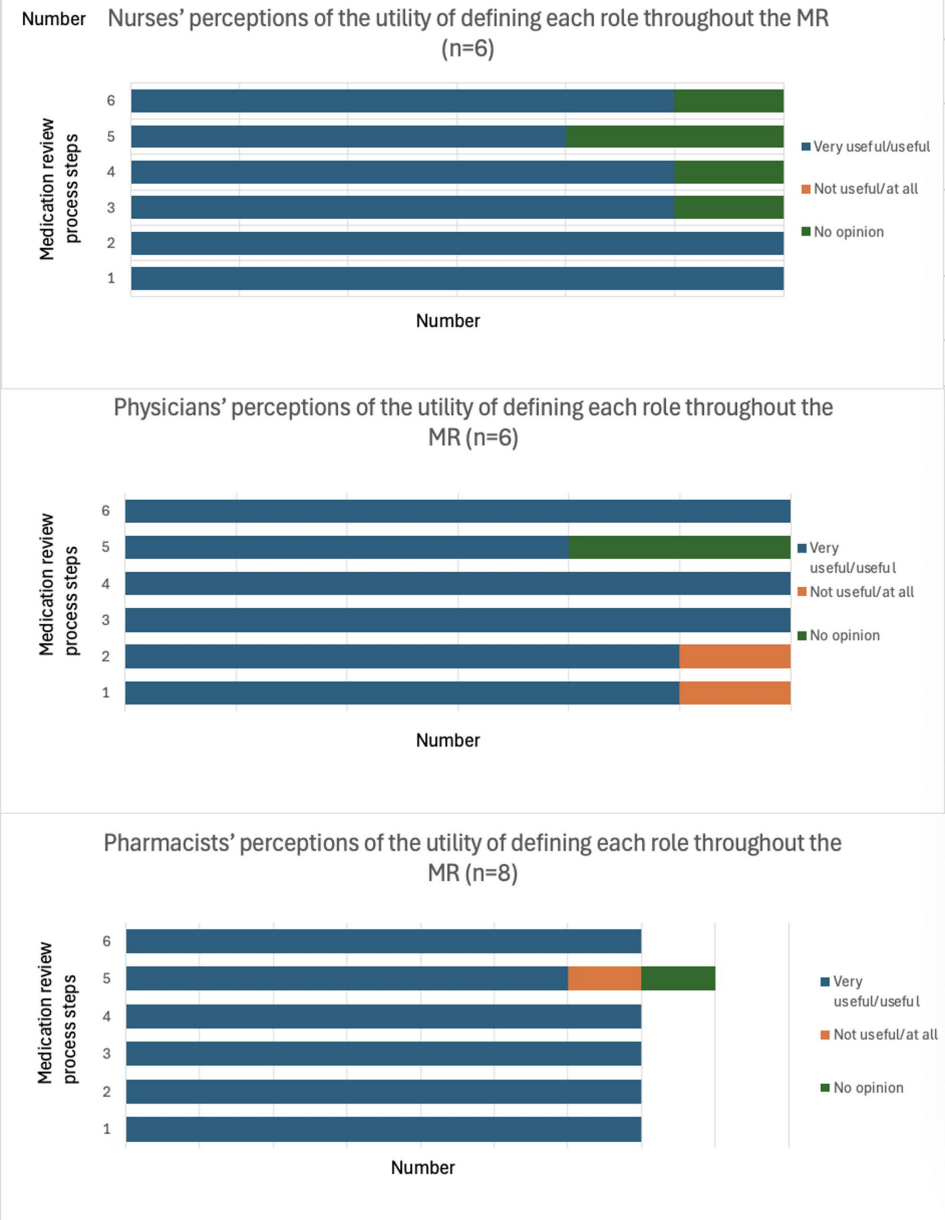
HCPs’ utility of defining each step of the process before its implementation. Step n°1 = Selection of residents, n°2 = data collection, n°3 = MR, n°4 = treatment plans, n°5 = resident/family involvement, n°6 = application and clinical follow-up

The utility of defining each step of the process before its implementation was recognized by most of the HCPs, as presented in Fig. 2.

**Fig. 3:**
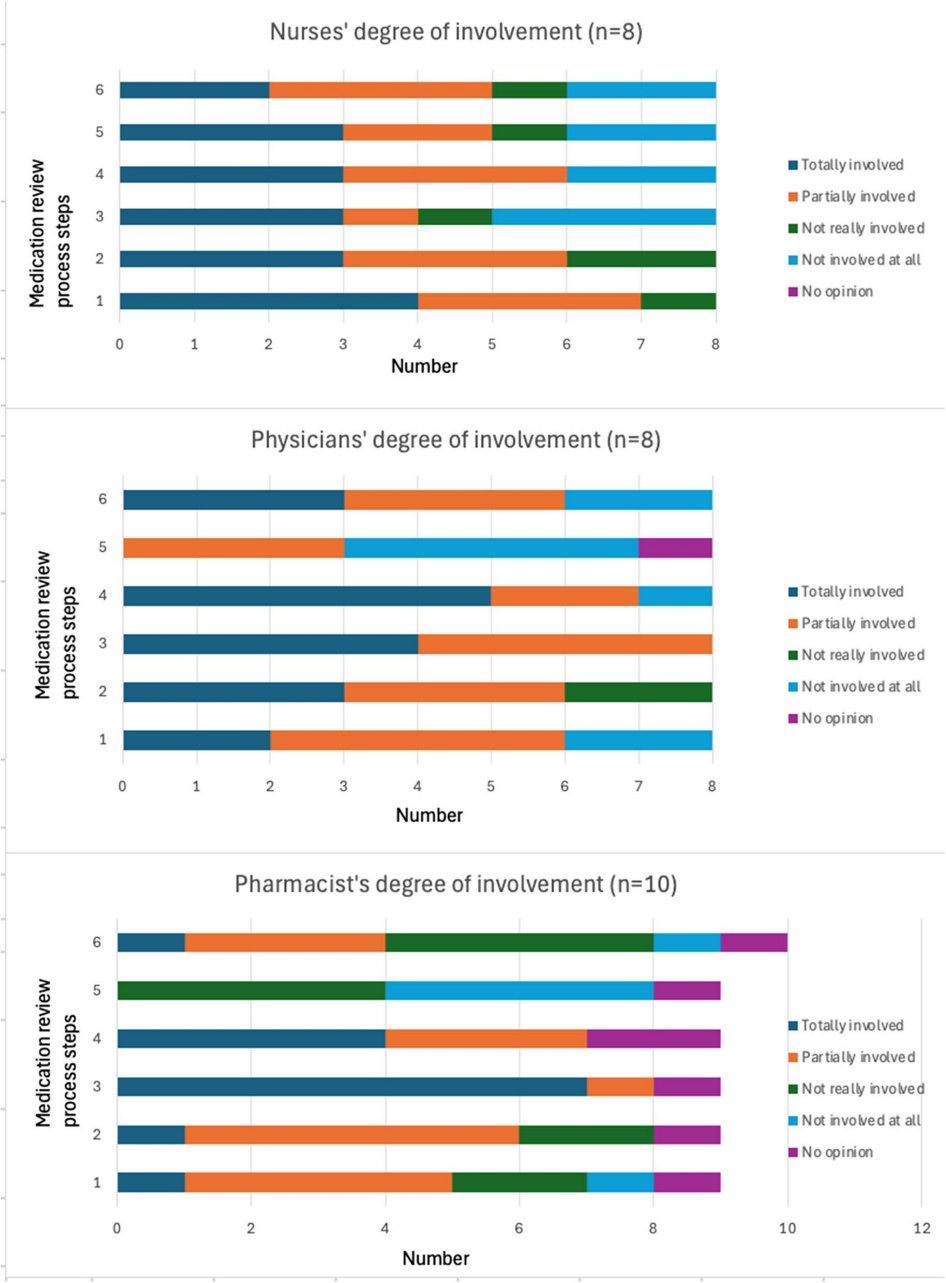
Degree of involvement by profession. Step n°1 = Selection of residents, n°2 = data collection, n°3 = MR, n°4 = treatment plan, n°5 = resident/family involvement, n°6 = application and clinical follow-up

Overall, participants reported a high level of involvement in all stages of the process, except for the involvement of residents and their families in the deprescribing process, expressed by physicians and pharmacists. The results are presented in Fig. 3.

During the focus groups and interviews, the pharmacists mentioned that they were not as involved as the nurses in the last two steps (involvement of residents and clinical follow-up). Table 2 details the time required for performing MRs.

**Table 2:** Time required for each step of the process for all the residents in one facility

MR process step	Time (minutes) required for each step of the MR process; mean (range)					
	1	2	3	4	5	6
Nurses	44 (20–60) *n* = 8	102 (0–240) *n* = 8	65 (0–180) *n* = 8	54 (0–180) *n* = 8	65 (0–180) *n* = 8	61 (0–180) *n* = 8
Physicians	12 (0–30) *n* = 8	21 (0–60) *n* = 8	26 (0–120) *n* = 8	12 (0–30) *n* = 8	11 (5–40) *n* = 8	18 (0–60) *n* = 8
Pharmacists Utility (*n* = 7)	10 (0–20) *n* = 9	31 (0–120) *n* = 9	161 (0–360) *n* = 9	52 (0–180) *n* = 9	2 (0–15) *n* = 9	15 (0–60) *n* = 9

*MR* Medication review*, Step n°1* Selection of residents*, n°2* data collection*, n°3* MR*, n°4* treatment plan*, n°5* Resident/family involvement*, n°6* application and clinical follow-up

#### Online platform for participating pharmacists

Regarding the utility of the platform, four of the ten pharmacists did not express an opinion, five claimed that it was not useful, and one mentioned that it was useful. They would have liked to exchange ideas with other participating pharmacists, but time constraints and technical problems related to access were cited as reasons for not using the platform. They suggested using a single platform to receive training materials and to exchange with other participants.

### Implementation outcomes

#### Participation rate

All 10 pharmacists involved in the study responded to both questionnaires. Nearly half of the participating physicians (10/21) and 11 of the 17 nurses completed the T4 questionnaires. The qualitative element of the study included seven pharmacists in a focus group, three physicians (two in an online group interview and one in an individual online interview), and five nurses (three in a focus group and two in individual interviews.)

### Preparation stage

#### Adoption

As targeted, 10 NHs volunteered to join the pilot project, which included 21 physicians, 17 nurses, and 10 pharmacists. All NH teams signed the agreement form between January and March 2021, except for one pharmacist who had not yet been hired by the pharmacy at the time of the pilot project. Another NH returned the signed agreement form in July 2021 due to the reorganization of the pharmacist’s staff and the referring pharmacist’s time constraints. The participating NHs were geographically distributed through the Canton of Vaud. Eight had a geriatric mission and two a psychogeriatric one. NH size varied (mean: 57 beds; range 24–124), and the mean number of active years in the integrated pharmacy service was nine (range 5–12). The pharmacists were mainly chief pharmacists (*n* = 7/10) and had an average of six years of collaboration with the NH (range: 0–13). Two additional pharmacists joined during the project to support the referring pharmacists. Only two pharmacists had prior training in performing MRs. Five pharmacists reported having received general training during the opportunities and limits of deprescribing for older people in nursing homes OLD-NH project [35]. The 10 participating physicians were all general practitioners who specialized in internal medicine. Most of the nurses were head nurses (*n* = 5) or unit head nurses (*n* = 3), but there was one clinical nurse, one nurse with no specific function, and one who did not give this information. The mean years of collaboration within the NH was 10 for physicians (range: 2–21) and five for nurses (range: 1–22). Most (10 nurses and eight physicians) reported having no prior training in MRs, although some (two nurses and seven physicians) reported having done MRs prior to the project.

#### Reach

In total, MRs were completely performed for 55 of the targeted 75 residents as the process had to be interrupted in three NHs. In the first one (three residents), the pharmacist had no motivation to develop new treatment plans due to physician unavailability. In the two others (11 and six residents involved, respectively), modifications suggested by pharmacists were never discussed with the other HCPs. The reasons for non-fully implementing MRNH mentioned by the pharmacists of the two first NHs were a lack of time due to activities related to the Covid pandemic. One NH mentioned that it had no prior experience of IPS and arrived mid-project as a reason for non-implementing the project.

#### Acceptability

Most of the pharmacists (*n* = 6/10) and physicians (*n* = 7/10) were very satisfied or satisfied with the service at follow-up. Interestingly, the pharmacists’ acceptance of the service increased between T0 and T4: At T0, five considered it acceptable, while at T4, five considered it very acceptable. The nurses, however, were less satisfied, with only three of 11 being very satisfied or satisfied at follow-up, while two were not satisfied at all.

#### Feasibility for pharmacists

In terms of skills, more than half of the pharmacists (*n* = 6/10) felt ready to perform MRs at the end of the three-day training workshop. In the T4 questionnaire, most of the pharmacists indicated that the entire project was unfeasible in terms of time and financial resources (Table 3). However, during the focus group, the pharmacists qualified these statements by conceding that with more practice and without the extra work caused by the project, MRs could be conducted more efficiently, for example, as DRP coding. Finally, most of the pharmacists agreed that the staff resources needed for delivering the service was reasonable.

**Table 3:** Feasibility measures in terms of time, financial resources, and staff after delivering the service

*n* = 8	Totally agree	Agree	Do not agree	Strongly disagree	No opinion
The amount of time required to implement the service is manageable	0	2	4	1	1
The financial resources needed to carry out the service are reasonable	0	2	4	0	2
The staff needed to carry out the service is reasonable	1	5	0	0	2
The amount of time to document the service is reasonable	0	1	3	0	4
The preparation for carrying out the service is reasonable	0	3	0	0	5

### Implementation

#### Adaptation of NH-specific processes

The NH teams were encouraged to individualize each step of the MR process, and they discussed and documented the operationalization thereof. Based on data from NH documents, follow-up questionnaires, and focus groups, the major adaptations made are detailed below:

##### Resident selection

Most NHs documented having adhered to the inclusion criteria established by the project (i.e., residents aged 75 and over, long stay, life expectancy estimated at over six months). According to the physicians, residents were selected based on their stability, the complexity of their pathologies, their life expectancy, their capacity for discernment, their date of entry into the NH, and the physicians’ relationships with the family/resident. During the focus groups, two physicians mentioned that a proportion of 10% of residents was adequate for MRs during the year, and during the physician’s visit was considered an appropriate time to conduct MRs; however, some HCPs suggested that the pharmacists should also be present.

##### Data collection for performing MRs

As planned, the pharmacists collected general (date of birth, entry date in the NH, life and treatment goals, etc.) and clinical information about the residents, mainly from the NH records, as needed to perform MRs. In addition, medication lists from the pharmacy were also collected, as pharmacists are responsible for dispensing medicines to NH residents.

During the focus groups, one pharmacist indicated that access to NH records allowed them to conduct MRs independently, while another stated that they preferred to ask a nurse to collect such data so that important points, such as resident’s preferences, could be included.

##### Conducting MRs

Most of the pharmacists (*n* = 6) planned to use the tool provided by the research team in combination with other literature resources to conduct MRs. This was confirmed during the focus groups, during which one pharmacist reported putting the contents of the tool onto another electronic support (OneNote), while another pharmacist reported having adapted the tool by simplifying it. Furthermore, one pharmacist mentioned using the tool as a guide to perform MRs without filling it in.

##### Treatment plan implementation

The NH teams defined treatment plans during medical visits, at regular interprofessional meetings, or at meetings specifically dedicated to the MRNH project. In one case, a pharmacist sent a proposed treatment plan to a physician via e-mail. The average duration of interprofessional discussion per treatment plan validation was 24 min (range: 15–40 min) according to the pharmacists’ follow-up questionnaires.

##### Involvement of NH resident/family in the MR process

Information was provided to the residents and/or their representatives mainly by nurses and physicians during NH visits. Validation of treatment modifications was performed during visits or, when relatives were involved, over the phone. The NH teams did not systematically ask the residents to validate the modifications of their medications, but 24 residents concerned by the 43 documented MRs validated at least one.

#### Barriers and facilitators to implementing the new service

Table 4 outlines the barriers and facilitators to implementing the service per profession, with specific determinants for each step of the MR process based on pharmacists'perspectives. All three groups of HCPs mentioned interprofessional collaboration and residents’ systematic review of medications as facilitators. Common barriers mentioned by pharmacists and nurses were staff turnover and internal reorganization during the project.

**Table 4:** Barriers and facilitators of the new MR service per profession and barriers and facilitators to implementing each step of the MR process per pharmacists

**Facilitators**	**Barriers**
Physicians, Pharmacists, and Nurses (overall perspective)	
• Interprofessional collaboration• Residents’ systematic review of medication	• Staff turnover• Internal reorganization during the project

**Facilitators**	**Barriers**
Physicians (overall perspective)	
• Safety of the MR process• Innovation of the project• Improvement for residents’ quality of life• New perspective on the medications of a resident	• Planning modifications once at a time• No additional process needed as deprescribing was already practiced before the project• Lack of objective criteria to select the type of residents who could benefit from a MR
Nurses (overall perspective)	
• Support from physicians and pharmacists• Reflections on medications	• Lack of time• Lack of follow-up from pharmacists• Lack of consideration for nurses’ organization• Few exchanges between residents, pharmacists, and physicians
Pharmacists (overall perspective)	
• Audit and feedback during the study• Patient-centered service• Physician enthusiasm• Clinical support• Use of their own tool to perform MRs• Validation of the process during the preparation phase	• Lack of involvement of physicians• Lack of time• Difficulty using the proposed tools
Pharmacists– by implementation step	
1. Selection of residents	
• Clear process pre-defined• Involvement of NH teams	• Little involvement from physicians• Residents selected by a single referring physician at the NH
2. Data collection	
• Complete access to the NHs’ residents’ records • Good relationship with the head nurse	• Missing data• Time needed for data collection
3. MRs	
• Training• Discussion with colleagues with a practice in MRs	• Discrepancy between the time taken to perform MRs versus the scheduled one• All MRs performed simultaneously
4. Treatment plans	
• Direct transcription of decisions in NH records during the interprofessional discussion• Involvement of physicians and nurses• Technical support from investigators	• Physicians’ willingness to stop several medications simultaneously• Refusal to deprescribe medications initiated by specialists
5. Patient/family involvement	
• Explanation to resident for consent	• Lack of communication between HCPs regarding each other’s roles in project implementation• Resident dependency
6. Application and clinical follow-up	
• Documentation provided	• Lack of involvement by nurses and pharmacists• Little interprofessional communication

*HCP* Healthcare professional, *MR* Medication review, *NH* Nursing home

Additional barriers and facilitators were identified through the interviews and focus groups. The definition of each professions’ role in the process of deprescribing prior to implementing the service was seen as a facilitator by both nurses and pharmacists. According to some nurses, physicians specializing in psychogeriatrics were particularly supportive of the MR process. Some nurses also mentioned the support of policymakers, the agreement of families, nurses’ internal organization, and the tools available to develop their clinical skills as facilitating factors. For the pharmacists, the implications of nurses and physicians, access to online resources, access to NH records (including resident history), and experiential learning were seen as facilitators.

In contrast, all three groups of HCPs saw the general work organization as a critical barrier to implementing the new service. For example, one nurse mentioned that the internal organization of the pharmacy, which involved two referring pharmacists, led to a lack of monitoring of the service. Time to perform and document the service was also cited as a barrier by all three groups of HCPs, while both pharmacists and physicians identified NHs’ psychogeriatric mission as a barrier due to the complexity of residents'medications. One physician mentioned the absence of more objective criteria to select residents who could benefit from an MR as a barrier; however, other physicians highlighted the flexibility of resident selection as a facilitator. The pharmacists found the innovation of the service, their own lack of experience, and the involvement of multiple prescribers, including specialists, as barriers. On their side, physicians mentioned the short follow-up time and the reappearance of symptoms after deprescribing as barriers. The nurses struggled with residents’ refusal of deprescribing, and some experienced the withdrawal of a physician from the project, which compromised the implementation of the service.

The HCPs suggested some improvements that could help address the barriers cited. For example, the nurses mentioned support measures for pharmacists to better coordinate the service, the organization of a meeting with a NH’s nursing staff and pharmacist to explain the project while raising awareness about medication inappropriateness, and the introduction of systematic MRs for all residents who join the NH. The pharmacists raised the idea of being mentored by experienced pharmacists, discussing the process or clinical issues with colleagues, and implementing one change of treatment at a time rather than several simultaneously. One physician mentioned the use of an automatic system to identify inappropriate drugs.

#### Fidelity

The average time for pharmacists (*n* = 8) to perform a MR was 181 min (SD: 97; range: 60–360). Only three pharmacists conducted the MRs within the average 120 min scheduled for the project. During the focus groups, the pharmacists expressed that MRs took more time than anticipated due to a lack of prior experience and the need for a change of practice. To encourage the implementation of this new service, they suggested having more practical tools, such as ready-to-use guidelines and more exchange opportunities among pharmacists.

Prior to the start of the project, eight of the 10 NHs had defined and documented their individualized processes and sent them to the research team; however, two NHs did it after starting the process. The remaining NH dropped out before the end of the project without transmitting their documents to the research team.

Due to the increased activities in community pharmacies related to the Covid pandemic, in addition to regular activities related to the existing pharmacy service, and the difficulty of planning meetings around summer vacations, six of the 10 NHs were late in the planned implementation schedule. However, seven of the 10 NHs reached the targeted number of MRs (i.e., 10% of their residents). The reasons mentioned by pharmacists for not reaching the required number were refusal of consent to transmit data by a resident’s relative and lack of time due to other activities. One pharmacist mentioned not having received the needed information from physicians and loss of motivation. In total, 43 treatment plans, created using the template provided by the research team, were transmitted for analysis.

#### Perspectives on maintenance

Based on the questionnaires, most of the nurses (*n* = 7/11), pharmacists (*n* = 7/10), and physicians (*n* = 7/10) would recommend that other NHs provide MR services. Moreover, seven of eight nurses, six of eight physicians, and six of eight pharmacists thought it would be useful to continue providing this service. According to the focus group, three pharmacists intended to continue MRs provided they were remunerated. Most of the HCPs recognized the importance of maintaining MRs on a routine basis but indicated that implementing this service in all NHs in the long term would require time. However, two physicians indicated that performing MRs on 10% of their residents on a routine basis, as planned in the pilot project, seemed feasible. According to the pharmacy service’s monitoring, seven of the 10 participating NHs continued providing the service in 2022.

#### Impact

The percentage of DRPs (*n* = 120) considered partially or completely resolved at follow-up was 82.5% (IC: 74.5–90.7%) according to the 43 treatment plans transmitted to the research team. Indeed, the lack of two transfer agreements, the loss of data from six NH residents due to the personal computer theft of a pharmacist and the death of four residents during the follow up, 12 MRs could not be analyzed. The mean number of DRPs per resident (*n* = 43) detected by pharmacists was 5.2 (SD 2.1). According to DRPs identified by pharmacists, 42% (of 229) were related to safety issues and 28% to effectiveness issues. As a result of interprofessional team discussions, 145 treatment modifications issued from the 229 propositions (63%) made by pharmacists to resolve DRPs were accepted by the NH team, and physicians in particular. The main reasons for non-implementing the propositions were patient refusal (5), refusal of specialist physicians (2), hospitalization of residents (2), unnecessary modifications following laboratory results (2), omissions or other selected propositions (6). Of these 145, 128 modifications (88%) were implemented, and 120 (83%) had been maintained at follow-up. The number and causes of DRPs identified are detailed in Table 5. The course of six treatment modifications led to the reintroduction of the initial medication due to the recurrence of symptoms. The other two reasons were because two residents refused to stop medication.

**Table 5:** Number and types of causes of drug-related problems identified by pharmacists

Description of causes of DRP	Number of causes of DRPs (*n* = 229)	Number of NHs concerned (*n* = 9)	Number of residents concerned (*n* = 43)
Inappropriate drug according to guidelines/formulary	63	7	33
No indication for drug	33	6	21
Drug dose of a single active ingredient too high	23	7	15
No or incomplete drug treatment despite existing indication	18	6	13
Duration of treatment too long	16	7	14
Inappropriate combination of drugs or drugs and herbal medications or drugs and dietary supplements	15	5	9
Too many different drugs/active ingredients prescribed for indication	14	5	9
Drug dose too low	12	5	11
Inappropriate duplication of therapeutic group or active ingredient	7	3	5
Inappropriate timing of administration or dosing intervals by a health professional	8	3	4
Inappropriate drug form/formulation (for this patient)	6	5	6
Dosage regimen too frequent	4	3	3
Dose timing instructions wrong, unclear or missing	3	3	4
Type of DRP missing	2	2	2
Necessary information nor provided or incorrect advice provided	1	1	1
Wrong drug or strength dispensed	1	1	1
Patient intentionally uses/takes less drugs than prescribed or does not take the drug at all for whatever reason	1	1	1
Patient decides to use unnecessary drug	1	1	1
Inappropriate timing or dosing intervals	1	1	1

*DRP* Drug related problems*, NH* Nursing home

Based on the questionnaires, six nurses (*n* = 6/9) estimated that the service had no effect on residents’ physical well-being, while three nurses believed that it had a positive impact. Concerning residents’ psychological well-being, most nurses (7/9) felt that it had no effect, while one nurse felt it had a positive one. Nevertheless, five felt that the service had a positive effect on residents’ state of awareness during the day, while four observed no effect in this regard. Finally, most of the nurses (6/9) felt that the service had a positive impact on the quality of the residents’ exchanges with others, while three found that it had no effect. Following the introduction of the service, most physicians felt that it had no effect on residents’ physical (6/9) or psychological well-being (7/9). However, the majority found that the service had a positive impact on residents’ state of awareness during the day (5/9) and on the quality of their exchanges with others (6/9).

At follow-up, 15 residents had fallen, four had been hospitalized, and physical restraints had been used on two, while four residents died before follow-up. According to the SAE reports, none of these deaths were related to deprescribing.

## Discussion

In general, the HCPs found the six implementation strategies proposed in the MRNH pilot project useful. However, there is a need to adapt some of them, including pharmacist training, financial incentives, and clinical support. Although monitoring the teams’ progress was considered useful, facilitation needs to be strengthened to improve service implementation. In particular, the results identified the utility of defining specific processes for each NH when implementing such services. The pharmacists indicated that a unique communication system would be an important element for future implementation. Performing high-quality medication reviews requires a solid training in clinical pharmacy, which cannot be fully acquired in only a few days of training. In our study, the three-day training session was not intended to provide comprehensive expertise in clinical pharmacy, but rather to offer a practical framework and methodological tools tailored to the specific context of the participating pharmacists, many of whom already had prior experience of medication management in nursing homes. Furthermore, the high acceptance and implementation rates can be explained by the fact that the collaborative process was already well established between pharmacists, nurses and physicians in the context studied, and the fact that the pharmacists who participated were experienced.

Near three-quarters of the targeted number of residents (73%) was reached and benefited from an MR (55/75). This result aligns with that of another study that found a reach rate of 63% in a community pharmacy setting [36]. The pharmacists considered the conduction of MRs for 10% of residents annually to be feasible in routine practice under certain conditions (e.g., with adequate financial incentives, adequate training, and guidelines). Pharmacists’ support is important, as MRs are mostly a pharmacist-led service.

As most HCPs found it useful to define each sub-process among themselves in advance, this should be encouraged in future implementation efforts. Furthermore, the analysis of barriers and facilitators encountered by the NHs yielded practical information related to the specific steps of the implementation process. Interprofessional collaboration and organization, as well as the definition of each professions’ role in the process of deprescribing prior to the implementation of the service, are critical. Many improvements were proposed by the HCPs, including the use of a software solution to identify inappropriate drugs; however, no consensus emerged. Interestingly, the physicians’ opinions on flexibility of resident selection were mixed. More generally, a certain heterogeneity of practices within NHs occurred at each sub-process. Not strictly defining each sub-process thus allowed the HCP teams to adapt to their internal contexts, which is also crucial for successful health service implementation [37].

Fidelity was considered low, as only three pharmacists performed MRs within the allocated time (two hours). In addition, the planned schedule to implement the service was not followed due to extra activities related to the Covid pandemic, which should not be an obstacle to future implementation. The time required to conduct each MR should be monitored to ensure that the estimated time (two hours) accurately reflects the average over the long term, considering the experience gained and excluding additional work related to the pilot project.

It is noteworthy that, from a logical perspective, given that MR step 3 was intended to be conducted exclusively by pharmacists, all nurses and physicians would have been required to respond within 0 minute to complete it. This could be indicative of either a misunderstanding regarding this step and the overall process or a consultation with colleagues by the pharmacists in the formulation of the proposals. Unfortunately, this inconsistency was not investigated further.

All participating NHs except one maintained the service in routine practice after the pilot project. However, as participants in the MRNH pilot project were early adopters, particular attention should be paid to future NHs that attempt implementation [38]. In accordance with the literature [28, 39], adequate financial incentives should be provided to all participating HCPs. The MRNH project highlighted the limited possibility for physicians to charge for services provided in the absence of patients (i.e., 30 min per three months). Thus, a 20-min fee for physicians will be included in the service, supported by the regional health authorities. The organization within an institution or interprofessional team (nurse–pharmacist–physician) has been found to be a determinant of the integration of this new service [40], which was also demonstrated in our results. Specifically, the involvement of physicians during the initial phase should be monitored to address potential barriers, as the current as well as previous studies [41, 42] have shown that this is pivotal to successfully completing the process. Adding facilitation, which can be defined as external support aimed at facilitating the implementation of an intervention by addressing barriers such as changing attitudes, habits, skills, and ways of thinking and working [43, 44], would help to identify which NH teams need specific support during service implementation and provide HCPs with practice-based insights from peers with which to overcome hurdles, as suggested by the interviewed HCPs.

The impact of the project was significant, with an 83% rate of DRP resolution; prior studies have shown rates ranging from 29–74% [45–47]. However, a certain risk of self-reporting bias exists, as DRP status at follow-up was documented by the same pharmacists who performed the MRs. The sample of the current study was limited in size and included early adopters [48, 49] with experience in resolving DRPs and/or implementing interprofessional services. Thus, the good rate of NHs which reached the targeted 10% of MRs may be attributed to the selection of highly motivated NHs adept of interprofessional medication optimization. Therefore, the results of this project should be considered with caution when designing the future dissemination of MR services.

The PCNE classification was used to identify the causes of DRP because it is a structured and detailed classification that is well suited to the international and Swiss context. The PharmDISC classification system (Pharmacists'Documentation of Interventions in Seamless Care) [50] could also have been used for its ease of application and its specificity of being well suited to collaborative environments such as primary care, NHs, or multidisciplinary consultations.

No safety issues emerged regarding the occurrences of falls, hospitalizations, or deaths, which were observed in the previous randomized controlled trial that took place in the same context [27]. The physicians’ perceptions of the service’s impact on residents diverged from the nurses’one to some extent, but a positive impact was frequently observed on the physical and psychological well-being of some residents, as well as on the quality of exchanges with them. Further evaluation should be conducted using, for example, patient-reported outcome measures [50] to better observe other potential effects (i.e., clinical, cognitive, and social effects) as well as medication safety and residents’ well-being. As residents in NHs often suffer from cognitive impairment, the feedback from their relatives in such evaluation should be considered. Medication reviews have been performed for 10% of a NH's residents, suggesting that the service will slowly target all residents. Therefore, a more effective strategy for this service would be to prioritize residents at higher risk of DRPs, such as those with polypharmacy, recent hospital discharges or specific comorbidities.

This study has several strengths, including the use of an implementation science framework as well as both quantitative and qualitative data sources to evaluate the implementation, factors, process, and strategies. Another strength is the real-world practice setting, which allowed for the feasibility and acceptability of the service in routine practice to be evaluated and the implementation strategies to be reinforced based on real needs reported by the HCPs. The analysis of the implementation process provides valuable information about contextual factors related to continuing to implement MRs in Swiss NHs.

This study also has several limitations. Regarding specific barriers to implementing the MR process, only the pharmacists’ perspectives were collected. This was done to avoid lengthening the questionnaire for other HCPs and because the service was mostly led by pharmacists. However, the pharmacists indicated that they were not as involved as the nurses in the resident involvement stage and clinical follow-up. Future studies could evaluate the barriers and facilitators of these two specific steps from the nurses’ perspective. All participating HCPs were able to give their perspectives on their involvement in the various stages of the service during the focus groups and interviews.

Another limitation was that the duration of each step of the MR process was reported by the HCPs at intervals of a few months, which may have led to reporting bias. This could have been avoided by conducting field observations. Finally, not all the data from the MRs and treatment plans could be retrieved by the research team due to two refusals, four deaths before the four-month follow-up, and six not being transmitted by the pharmacist after the loss of their personal computer. Finally, only a small number of DRPs were evaluated, which may have impacted the accuracy of the values obtained. This underscores the importance of continuing this experiment and its follow-up.

## Conclusion

The implementation of MRs mostly led by community pharmacists in NHs was attained and accepted by the participating interprofessional teams, who recommended the dissemination of the service and maintained it in their NHs. The impact on DRPs is important and may benefit residents’ physical and mental well-being. After having experienced the MR process, the HCPs suggested some improvements that could be made to the implementation strategies to support a scaling-up, for example, adding a specific remuneration for physicians, adjusting training content, reinforcing facilitation, clinical support, and setting up one information and a unique communication system. The results of the current study support the regional health department's decision to extend the service to more NHs in the future. The latter consists of a federal quality of care project funded over 3 years to support the development of informed implementation strategies in the field, and to extend the MRs service to NHs in other cantons.

## Supplementary Information

Supplementary Material 1.

Supplementary Material 2.

Supplementary Material 3.

Supplementary Material 4.

Supplementary Material 5.

Supplementary Material 6.

Supplementary Material 7.

## Data Availability

The datasets generated during and analysed during this study are available from the corresponding author upon reasonable request.

## Abbreviations

DRPs: Drug Related Problems
FISpH: Framework for the Implementation of Pharmacy Services
HCPs: Healthcare professionals
MRNH: Medication Reviews in Nursing homes
NH: Nursing home
PIMs: Potentially inappropriate medications
RE-AIM: Reach, Effectiveness-Adoption, Implementation, Maintenance framework
