# Supplementary material for: Implementation of medication reviews to optimize the use of medications in Swiss nursing homes: a mixed-methods study
Source: BMC Health Serv Res. 2025 Jul 8;25:943. doi: 10.1186/s12913-025-13042-8 (PMC12239413; doi:10.1186/s12913-025-13042-8)
Supplement: Supplementary file 1 — Supplementary Material 1. [file 12913_2025_13042_MOESM1_ESM.docx]

**Pharmacist T0** Nursing Home Code: _______Date: ________

This evaluation aims to gather your opinion on the training you attended as part of the MRNH project and the proposed medication review (MR) process. It is a qualitative assessment of the usefulness of the training and process for conducting MRs, without right or wrong answers. Please complete this questionnaire as accurately as possible based on your perspective.

**PREVIOUS EXPERIENCE**

**A.1** Since what year have you personally been collaborating with this nursing home? ________

**A.2** What is your position?
□ Chief Pharmacist
□ Assistant Pharmacist
□ Other: Please specify ______________________

**A.3** Have you previously attended training on medication reviews (excluding the MRNH project training)?
□ Yes
□ No
If yes, in what context? __________________________________________________________________________________

**A.4** Have you previously attended training on deprescribing?
□ Yes
□ No
If yes, in what context?
□ QC-Demo and/or IDeI study
□ Other: Please specify ______________________

**A.5** Have you previously conducted medication reviews in your nursing home before the MRNH project?
□ Yes
□ No
If yes, how many resident cases have you reviewed? ______ residents/year for ___ years.
In what format do you conduct these reviews?
□ Quick medication review at admission
□ Upon request from nursing or medical staff
□ Systematic review (e.g., once per year for each resident)
□ Structured medication review: Specify timing and frequency ___________________________________________
□ Other: Please specify ______________________

**MRNH TRAINING**

**B.1** Who initiated the decision to participate in the pilot project?
□ Pharmacist
□ Physician
□ Nursing team
□ Nursing home management
□ Other: Please specify ______________________

**B.2** On a scale of 0 to 5 (0 = minimum), how would you rate the overall quality of the training you received for conducting medication reviews in nursing homes? □ 0 □ 1 □ 2 □ 3 □ 4 □ 5

**B.3** Please check one box per statement below that best applies:

| **After the MRNH training, ...** | **Strongly Agree** | **Agree** | **Neutral** | **Disagree** | **Strongly Disagree** | |
| --- | --- | --- | --- | --- | --- | --- |
| I feel ready to conduct medication reviews. | □ | □ | □ | □ | □ |  |
| The medication review tool seems easy to use. | □ | □ | □ | □ | □ |  |
| The treatment modification plan seems easy to use. | □ | □ | □ | □ | □ |  |
| The training content was sufficient for conducting medication reviews. | □ | □ | □ | □ | □ |  |
| The exercises provided during the training were sufficient for conducting medication reviews. | □ | □ | □ | □ | □ |  |
| The training overall (content + exercises) met my expectations for conducting medication reviews. | □ | □ | □ | □ | □ |  |

**B.4** Evaluate the usefulness of the different topics covered in the MRNH training:

| Training Topics | Very Useful | Useful | Neutral | Not Useful | Not at All Useful |
| --- | --- | --- | --- | --- | --- |
| Clinical reasoning in geriatrics | □ | □ | □ | □ | □ |
| Conducting a medication review in nursing homes | □ | □ | □ | □ | □ |
| Interprofessional communication techniques (incl. workshop) | □ | □ | □ | □ | □ |
| Presentation of the pilot project (general, process, data collection) | □ | □ | □ | □ | □ |

**B.5** In your opinion, should this training be offered to physicians and nurses involved in the project?
□ Yes □ No □ Unsure
If yes, which part(s) of the training would you suggest for them?
□ Clinical reasoning in geriatrics
□ Conducting a medication review in nursing homes
□ Interprofessional communication
□ Project process

**B.6** What are the two strengths of the training you received?

**B.7** What are the two weaknesses of the training you received?

**B.8** Comments and suggestions regarding the MRNH training:

**MEDICATION REVIEW PROCESS**

**C.1** On a scale of 0 to 5 (0 = minimum), how would you rate the proposed process in the MRNH project?
□ 0 □ 1 □ 2 □ 3 □ 4 □ 5

**C.2** Overall, are you satisfied with the process in the MRNH pilot project?
□ Very satisfied □ Satisfied □ Neutral □ Dissatisfied □ Very dissatisfied

**C.3** Comments and suggestions regarding the medication review process:

**OTHER COMMENTS, REMARKS, GENERAL SUGGESTIONS:**

Thank you for completing this questionnaire!
Feel free to provide additional feedback throughout the project at ddp.emsvd@unisante.ch. The new practice evaluated in this project should make sense in practice, so your input is essential!
