# Supplementary material for: Implementation of medication reviews to optimize the use of medications in Swiss nursing homes: a mixed-methods study
Source: BMC Health Serv Res. 2025 Jul 8;25:943. doi: 10.1186/s12913-025-13042-8 (PMC12239413; doi:10.1186/s12913-025-13042-8)
Supplement: Supplementary file 2 — Supplementary Material 2. [file 12913_2025_13042_MOESM2_ESM.docx]

**Pharmacist T4** Nursing Home Code: ________ Date: ________

This evaluation aims to gather your opinion on the MRNH project in which you participated. There are no right or wrong answers. Please complete this questionnaire as accurately as possible based on your experience.

1. **IMPLEMENTATION STRATEGIES EVALUATION :**

**A1.** MRNH Training Adequacy: Do you have any comments regarding the adequacy of the MRNH training for conducting medication reviews?_____________________________________________________________________________________________________________

| **A.2 At the end of the MRNH pilot project, I estimate:** | **Strongly Agree** | **Agree** | **Neutral** | **Disagree** | **Strongly Disagree** |
| --- | --- | --- | --- | --- | --- |
| I was able to conduct medication reviews easily. | □ | □ | □ | □ | □ |
| The medication review tool is useful. | □ | □ | □ | □ | □ |
| The medication review tool is easy to use. | □ | □ | □ | □ | □ |
| The treatment plan is adequate. | □ | □ | □ | □ | □ |
| The training received was sufficient for conducting medication reviews. | □ | □ | □ | □ | □ |

**A.3** Have you reached the number of medication reviews requested as part of the MRNH project (10% of your nursing home's residents)?

□ Yes □ No

**A.4** Were you able to define the various stages of the project (implementation process) as an interprofessional team,before starting the project?

**A5. Description of implementation processes**

| **A5.1 How useful was it for you to describe and define the implementation processes before carrying out the medication reviews?** | **Very useful** | **Useful** | **Neutral** | **Not useful** | **Not at all useful** |
| --- | --- | --- | --- | --- | --- |
| 1. Selection of residents | □ | □ | □ | □ | □ |
| 2. Data collection for medication review | □ | □ | □ | □ | □ |
| 3. Conducting medication reviews | □ | □ | □ | □ | □ |
| 4. Defining treatment modification plans | □ | □ | □ | □ | □ |
| 5. Involvement of residents/families | □ | □ | □ | □ | □ |
| 6. Monitoring modifications and documentation | □ | □ | □ | □ | □ |
| 7. Data collection and transmission to Unisanté | □ | □ | □ | □ | □ |

| **A.6** On a scale of 1 to 5, how do you evaluate the application of your processes based on defined criteria? (1 = minimum, 5 = maximum)  **A.7** What facilitated their application? What prevented their application? | **1** | | **2** | | **3** | | **4** | | **5** |  |
| --- | --- | --- | --- | --- | --- | --- | --- | --- | --- | --- |
| 1. Selection of residents | □ | | □ | | □ | | □ | | □ |  |
| What factors facilitated the application of these processes? | | | | | | | | | | |
| 2. Data collection for medication review | □ | | □ | | □ | | □ | | □ |  |
| What factors facilitated the application of these processes? | | | | | | | | | | |
| 3. Conducting medication reviews | □ | | □ | | □ | | □ | | □ |  |
| What factors facilitated the application of these processes? | | | | | | | | | | |
| 4. Defining treatment modification plans | | □ | | □ | | □ | | □ | □ | |
| What factors facilitated the application of these processes? | | | | | | | | | | |
| 5. Involvement of residents/families | | □ | | □ | | □ | | □ | □ | |
| What factors facilitated the application of these processes? | | | | | | | | | | |
| 6. Monitoring modifications and documentation | | □ | | □ | | □ | | □ | □ | |
| What factors facilitated the application of these processes? | | | | | | | | | | |
| 7. Data collection and transmission to Unisanté | | □ | | □ | | □ | | □ | □ | |
| What factors facilitated the application of these processes? | | | | | | | | | | |

**A.8** Among the following questions:

A.8.1 How useful was the follow-up conducted by Unisanté (explanations, answers to questions, availability, etc.) for conducting medication reviews? □ Very useful □ Useful □ Neutral □ Not useful, □ Not at all useful

A.8.2 How useful was the exchange forum for conducting medication reviews?

□ Very useful □ Useful □ Neutral □ Not useful, □ Not at all useful

A.8.3 How useful was the clinical coaching from Unisanté’s pharmaceutical assistance for conducting medication reviews? □ Very useful

□ Useful □ Neutral □ Not useful, □ Not at all useful

1. **MEDICATION REVIEW SERVICE**

**B.1** On a scale of 1 to 5, to what extent do you consider medication review to be a good way of optimizing medication for your nursing home residents? (1=minimum, 5=maximum)

**B.2** To what extent do you consider the evidence for the new practice to be solid? □ Strongly agree, □ Agree □ Neutral, □ Disagree, □ Strongly disagree

| **B.3**  How involved were you in each stage of the process for all residents in your nursing home? | Totally involved | Slightly involved | Not really involved | Not at all involved | No opinion |
| --- | --- | --- | --- | --- | --- |
| 1. Selection of residents | □ | □ | □ | □ | □ |
| 2. Data collection for medication review | □ | □ | □ | □ | □ |
| 3. Conducting medication reviews | □ | □ | □ | □ | □ |
| 4. Defining treatment modification plans | □ | □ | □ | □ | □ |
| 5. Involvement of residents/families | □ | □ | □ | □ | □ |
| 6. Monitoring modifications and documentation | □ | □ | □ | □ | □ |
| 7. Data collection and transmission to Unisanté | □ | □ | □ | □ | □ |

**B.4** Can you estimate how much time you needed to complete each step for all the residents in your nursing home? (minutes)

Resident selection:____, Data collection for medication reviews:____, Conducting medication reviews:____, Defining treatment plans:____, Involving residents/relatives:____, Monitoring modifications and documentation:____

| **FEASIBILITY**  **B.5** To what extent do you agree with the following statements? | **Strongly Agree** | **Agree** | **Neutral** | **Disagree** | **Strongly Disagree** |
| --- | --- | --- | --- | --- | --- |
| The time required to implement this service was manageable | □ | □ | □ | □ | □ |
| The financial resources required to implement this service are reasonable |  |  |  |  |  |
| The personnel required to implement this service are reasonable | □ | □ | □ | □ | □ |
| The preparation for implementing this service is reasonable. | □ | □ | □ | □ | □ |

**B.6** How long, on average, did interdisciplinary discussions take to validate patient treatment plans (minutes per resident)?__________________

**B.7** On a scale of10 to 5, how do you rate the degree of involvement of nurses in implementing treatment modifications and follow-up measures in practice? (1= not at all involved∙e 5= totally involved)? □ 1 □ 2 □ 3 □ 4 □ 5

**B.8** On a scale of 1 to 5, how do you rate the degree of involvement of physicians in the completion of medication reviews in practice? (1= not at all involved∙e 5= totally involved)? □ 1 □ 2 □ 3 □ 4 □ 5

**B.9** Do you think it is useful to continue this type of practice in the future? □ Yes □ No □ No opinion

**B.10** Would you recommend other nursing home facilities to engage in a similar approach? □ Yes □ No □ No opinion

**B.11** - In your opinion, what are the two main strengths of this medication review process in nursing homes?

**B.12** In your opinion, what are the two main weaknesses of this medication review process in nursing homes?

**B.13** Overall, are you satisfied with the medication review service as proposed in the MRNH pilot project? □ Very satisfied □ Satisfied □ Neutral □ Slightly dissatisfied □ Not at all satisfied

C. Comments & Suggestions

Do you have any comments or suggestions?

Thank you for completing this questionnaire! Feel free to provide additional feedback throughout the project. The new process evaluated in this project must be meaningful for practice, so your opinion is essential!
