# Supplementary material for: Implementation of medication reviews to optimize the use of medications in Swiss nursing homes: a mixed-methods study
Source: BMC Health Serv Res. 2025 Jul 8;25:943. doi: 10.1186/s12913-025-13042-8 (PMC12239413; doi:10.1186/s12913-025-13042-8)
Supplement: Supplementary file 4 — Supplementary Material 4. [file 12913_2025_13042_MOESM4_ESM.docx]

**Physician** Nursing Home Code: ________ Date: ________

This evaluation aims to gather your opinion on the MRNH project in which you participated. There are no right or wrong answers. Please complete this questionnaire as accurately as possible based on your experience.

**PREVIOUS EXPERIENCE**

**A.1** Since what year have you been working in this nursing home? ________

**A.2** How many residents do you currently follow in this nursing home? ________

**A.3** Have you previously attended training on medication reviews?□ Yes □ No; If yes: Could you please specify which training.____________

**A.4** Have you previously conducted medication reviews in your nursing home before the MRNH project? □ Yes □ No If no, proceed to section B.

**A.4.1** In what format do you conduct these reviews?n□ Quick medication review at admission, □ Systematic review (e.g., once per year for each resident), □ Structured medication review as proposed in the MRNH project □ Other: Please specify________________

**A.4.2** Who else is generally involved in these reviews? □ Nurses, □ Pharmacists, □ Residents, □ Residents' families, □ Specific residents, □ Case-by-case basis, □ Other: Please specify: ____

**A.4.3** How many residents are involved? ______ residents/year.

**A5.** What type of medical specialization do you have? □general internal medicine, □ psychiatry, □ Other: Please specify: ____

**MEDICATION REVIEW**

**B.1** Would you have found it useful to participate in a 1-hour online information session about the project at the beginning?
□ Yes □ No □ No opinion

**B.2** Were you able to define the various stages of the project (implementation process) as an interprofessional team before starting the project? □ Yes □ No

**B.3** To what extent did you find it useful to define the following implementation processes before starting the project?

| **Process** | **Very Useful** | **Useful** | **Neutral** | **Not Useful** | **Not at All Useful** |
| --- | --- | --- | --- | --- | --- |
| Resident selection | □ | □ | □ | □ | □ |
| Data collection for medication reviews | □ | □ | □ | □ | □ |
| Conducting medication reviews | □ | □ | □ | □ | □ |
| Defining treatment modification plans | □ | □ | □ | □ | □ |
| Involving residents/relatives | □ | □ | □ | □ | □ |
| Monitoring modifications and documentation | □ | □ | □ | □ | □ |

**B.4** What selection criteria (other than the minimal inclusion criteria) have you chosen to select your residents who have benefited from a medication review?___________________________________________________________________________________

**B.5** **In your opinion, what impact did this approach have on the resident?**

- physical well-being (pain, functional autonomy, ability to move)? □ positive □ no effect □ negative □ no opinion

- psychological well-being (morale, mood, anxiety)? □ positive □ no effect □ negative □ no opinion

- the quality of exchanges during your visits or phone calls (presence, interactivity, consistency)? □ positive □ no effect □ negative □ no opinion

- How alert are you during the day (dynamism, energy)? )? □ positive □ no effect □ negative □ no opinion

**B.6** On a scale of 1 to 5 (1 = minimum), to what extent do you think the MRNH project benefited residents? □ 0 □ 1 □ 2 □ 3 □ 4 □ 5

**B.7** On a scale of 1 to 5, to what extent do you consider medication review to be a good way of optimizing the medication of your NH residents?(1=minimum, 5=maximum)

**B.8** To what extent do you consider the evidence for the new practice to be solid? □ Strongly agree, □ Agree □ Neutral, □ Disagree, □ Strongly disagree

| **B.9**  How involved were you in each stage of the process for all residents in your nursing home? | Totally involved | Slightly involved | Not really involved | Not at all involved | No opinion |
| --- | --- | --- | --- | --- | --- |
| 1. Selection of residents | □ | □ | □ | □ | □ |
| 2. Data collection for medication review | □ | □ | □ | □ | □ |
| 3. Conducting medication reviews | □ | □ | □ | □ | □ |
| 4. Defining treatment modification plans | □ | □ | □ | □ | □ |
| 5. Involvement of residents/families | □ | □ | □ | □ | □ |
| 6. Monitoring modifications and documentation | □ | □ | □ | □ | □ |

**B.10** On a scale of 0 to 5 (0 = minimum, 5 = maximum), how would you rate the involvement/support of the nursing team in implementing treatment modifications and follow-up measures? □ 0 □ 1 □ 2 □ 3 □ 4 □ 5

**B.11** How would you rate the involvement/support of the pharmacist in conducting medication reviews? □ 0 □ 1 □ 2 □ 3 □ 4 □ 5

**B.12** Can you estimate how much time you needed to complete each step for all the residents in your nursing home? (minutes)

Resident selection:____, Data collection for medication reviews:____, Conducting medication reviews:____, Defining treatment plans:____, Involving residents/relatives:____, Monitoring modifications and documentation:____

**B.13** In your opinion, how valuable is the interprofessional approach of this process?
□ Very useful, □ Useful, □ Neutral, □ Not useful, □ Not at all useful

**B.14** Do you think this type of practice should continue in the future?
□ Very useful, □ Useful, □ Neutral, □ Not useful, □ Not at all useful

**B.15** Would you recommend other nursing homes to participate in a similar initiative? □ Yes □ No □ No opinion

**B.16** Do you believe that your activities related to medication reviews are adequately covered by your current remuneration?
□ Yes □ No If no, what should your remuneration be per medication review? _______ CHF

**B.17** What do you consider to be the two strengths of this new medication review practice in nursing homes?

**B.18** What do you consider to be the two weaknesses of this new medication review practice in nursing homes?

**B.19** Overall, are you satisfied with the service as implemented in the MRNH pilot project?
□ Very satisfied, □ Satisfied, □ Neutral, □ Dissatisfied, □ Very dissatisfied

**C. COMMENTS, SUGGESTIONS:**

Do you have any comments or suggestions?

Thank you for completing this questionnaire! Feel free to provide additional feedback throughout the project. The new process evaluated in this project must be meaningful for practice, so your opinion is essential!
