# Supplementary material for: Implementation of medication reviews to optimize the use of medications in Swiss nursing homes: a mixed-methods study
Source: BMC Health Serv Res. 2025 Jul 8;25:943. doi: 10.1186/s12913-025-13042-8 (PMC12239413; doi:10.1186/s12913-025-13042-8)
Supplement: Supplementary file 5 — Supplementary Material 5. [file 12913_2025_13042_MOESM5_ESM.docx]

General Objectives:

- Identify priorities for quality improvement of the intervention in view of its future dissemination: What are the key elements that emerge from this focus group for successful implementation? Does one practice appear more favorable than another?

- Identify potential, innovative solutions to help future participants implement and evaluate these solutions.

At the start of the focus group, preliminary results from the questionnaire were presented to the healthcare providers, such as barriers and facilitators to service implementation.

| **Dimension** | Specific objective | Questions |
| --- | --- | --- |
| **Experience Sharing** | To gather participants' opinions on the results and experience sharing of the intervention. | - What do you think of these results? - To what extent do these results reflect what you expected? - Present the strengths and weaknesses. **Follow-up Questions:** - How did it go in your nursing home? To what extent were your processes defined differently as planned? |
| **Barriers and Facilitators** | To define the success and failure factors in implementing the new practice. | Barriers: - What difficulties did you encounter during implementation?  - What prevented the implementation of medication reviews?  - What are the main obstacles to implementing medication reviews in your nursing home?  Facilitators: - What are the key elements for implementing medication reviews routinely? - In your opinion, what are the important elements to put in place to ensure that medication reviews are effectively implemented? - What facilitated the implementation of medication reviews? |
| **Maintenance** | To test whether the new practice has been established as routine and/or institutionalized. | - To what extent have you been able to continue conducting standard medication reviews following the project? - For those who have not yet completed the project, to what extent have there already been discussions about the willingness to continue conducting medication reviews routinely? - To what extent do you consider it possible to integrate medication reviews into routine practice under current conditions (time, resources, coordination, selection of residents)? - What adaptations should be made compared to the pilot project to facilitate implementation in practice? - What would you recommend to a nursing home that wants to start? |
