# Supplementary material for: Implementation of medication reviews to optimize the use of medications in Swiss nursing homes: a mixed-methods study
Source: BMC Health Serv Res. 2025 Jul 8;25:943. doi: 10.1186/s12913-025-13042-8 (PMC12239413; doi:10.1186/s12913-025-13042-8)
Supplement: Supplementary file 6 — Supplementary Material 6. [file 12913_2025_13042_MOESM6_ESM.docx]

General Objectives:

- Identify priorities for quality improvement of the intervention in view of its future dissemination: What are the key elements that emerge from this focus group for successful implementation? Does one practice appear more favorable than another?

- Identify potential, innovative solutions to help future participants implement and evaluate these solutions.

At the start of the focus group, preliminary results from the questionnaire were presented to the healthcare providers, such as barriers and facilitators to service implementation.

| **Dimension** | Specific objective | Questions |
| --- | --- | --- |
| **Experience Sharing** | To gather participants' opinions on the results and experience sharing of the intervention. | - What do you think of these results? - To what extent do these results reflect what you expected? - Present the strengths and weaknesses. **Follow-up Questions:** - To what extent did this new service take up your time? - How do you assess the quality of the resident tracking document? - How did you find the involvement of physicians and pharmacists in the project?  **Additional Follow-up Questions:** - What is the interest, in your opinion, of a medication review service such as the one proposed in the MRNH pilot project? - What was the effect of the service on the resident? - In which domain did this service have a potentially positive effect? |
| **Barriers and Facilitators** | To define the success and failure factors in implementing the new practice. | **Barriers:** - What difficulties did you encounter during implementation? - What prevented the implementation of medication reviews? - What are the main obstacles to implementing medication reviews in your nursing home? **Facilitators:** - What are the key elements for implementing medication reviews routinely? - In your opinion, what are the important elements to put in place to ensure that medication reviews are effectively implemented? - What facilitated the implementation of medication reviews? |
| **Maintenance** | To test whether the new practice has been established as routine and/or institutionalized. | - To what extent have you been able to continue conducting regular medication reviews for the residents you follow in your nursing home? - To what extent do you find it useful to maintain a service such as medication reviews for your nursing home residents? - How do you think you can integrate this new service into the routine under the current conditions of the nursing home? - What would you recommend to another nursing home that wants to start? |
