# Supplementary material for: Implementation of medication reviews to optimize the use of medications in Swiss nursing homes: a mixed-methods study
Source: BMC Health Serv Res. 2025 Jul 8;25:943. doi: 10.1186/s12913-025-13042-8 (PMC12239413; doi:10.1186/s12913-025-13042-8)
Supplement: Supplementary file 7 — Supplementary Material 7. [file 12913_2025_13042_MOESM7_ESM.docx]

General Objectives:

- Identify priorities for quality improvement of the intervention in view of its future dissemination: What are the key elements that emerge from this focus group for successful implementation? Does one practice appear more favorable than another?

- Identify potential, innovative solutions to help future participants implement and evaluate these solutions.

At the start of the focus group, preliminary results from the questionnaire were presented to the healthcare providers, such as barriers and facilitators to service implementation.

| **Dimension** | Specific Objective | Questions / Items |
| --- | --- | --- |
| **Experience Sharing** | To gather participants' opinions on the results and experience sharing of the intervention. | - Present the strengths and weaknesses.   **Weak Points:**  - Few patients included.  - Side effects appeared after deprescription.  - Cases not complex enough.  - No objective criteria to select the type of resident who can benefit from a change.  - More pragmatic than evidence-based approach.  - Planning changes over time.  **Strengths:**  - Improved quality and effectiveness of treatment.  - Generally positive impact on the resident's condition.  - Reduction in the number of medications.  - Safety.  - Modern approach.  - Interprofessional collaboration.  - Reevaluation of medication use. **- What do you think of these results? - To what extent do these results reflect what you expected?** |
| **Maintenance** | To test whether the new practice has been established as routine and/or institutionalized. | - To what extent have you been able to continue conducting regular medication reviews following the project? - To what extent do you consider it possible to integrate medication reviews into routine practice under current conditions? (time, resources, coordination, selection of residents) - What adaptations should be made compared to the pilot project to facilitate implementation in practice? - What would you recommend to another nursing home that wants to start? |
| **Barriers and Facilitators** | To define the success and failure factors in implementing the new practice. | **Barriers:** - What difficulties did you encounter during implementation? - What prevented the implementation of medication reviews? - What are the main obstacles to implementing medication reviews in your Nursing home? **Facilitators:** - What are the key elements for implementing medication reviews routinely? - In your opinion, what are the important elements to put in place to ensure that medication reviews are effectively implemented? - What facilitated the implementation of medication reviews? |
